# Patterns of inpatient antibiotic use and antimicrobial resistance in the surgical wards of a Ugandan tertiary hospital: A mixed methods study

**DOI:** 10.1371/journal.pone.0352983

**Published:** 2026-07-24

**Authors:** Fiona Mutesi Magololo, Dathan Byonanebye, Flavia Dhikusooka, Adelline Twimukye, Doreen Agaba, Ellon Twinomuhwezi, Hope Mackline, Edith Namakula, Alex Emmanuel Elobu, Norbert Orwotho Tholith, Francis Kakooza, Andrew Kambugu, Henry Kajumbula

**Affiliations:** 1 Department of Medical Microbiology, School of Biomedical Sciences, College of Health Sciences, Makerere University, Kampala, Uganda; 2 Department of Research, Infectious Diseases Institute, College of Health Sciences, Makerere University, Kampala, Uganda; 3 Department of Global Health Security, Infectious Diseases Institute, College of Health Sciences, Makerere University, Kampala, Uganda; 4 School of Public Health, College of Health Sciences, Makerere University, Kampala, Uganda; 5 Directorate of Surgery, Mulago National Referral Hospital, Kampala, Uganda; Debre Markos University, ETHIOPIA

## Abstract

**Background:**

Inappropriate antibiotic use in surgical wards is a major driver of antimicrobial resistance (AMR), especially in low- and middle-income countries, where empirical prescribing is common. This study assessed inpatient antibiotic use and AMR patterns, integrating these findings with qualitative in-depth interviews (IDIs) which explored determinants of prescribing practices.

**Methods:**

A convergent mixed methods study design was employed on the surgical wards of Mulago National Referral Hospital (MNRH) in Uganda. Quantitative data was collected through Point Prevalence Surveys (PPS) of antibiotic use and retrospective analysis of routine laboratory AMR data. Qualitative data was obtained through IDIs with healthcare workers (HCWs). Quantitative data was analysed using R version 4.2.2 and STATA 19. In-depth interviews were recorded and transcribed verbatim, open coded and then analysed using NVivo 12.

**Results:**

Of 303 total surgical inpatients, 193/303 (63.7%) (95% CI: 58.0–69.12) were receiving at least one of the 281 antibiotic prescriptions and were included in this study. Surgical antibiotic prophylaxis (SAP) accounted for 256/281 (91.1%) of all antibiotic prescriptions, with 239/256 (93.4%) SAP extending beyond the first 24 hours. Ceftriaxone 89/281 (31.7%) (95% CI: 26.27–37.46), metronidazole 66/281 (23.5%) (95% CI: 18.66–28.89) and levofloxacin 24/281 (8.5%) (95% CI: 11.61–20.44) were most prescribed antibiotic agents. Watch category antibiotics were most used 152/281 (54.1%) (95%: 48.08–60.08), followed by Access category 129/281 (45.9%) (95% CI: 39.9–51.9). Although HCWs demonstrated awareness of AMS principles, formal stewardship systems were fragmented, and prescribing behaviour was strongly influenced by laboratory constraints, infection prevention and control (IPC) gaps and systemic challenges.

**Conclusion:**

Prolonged SAP and Watch category predominant prescribing occur within a context of high AMR prevalence and constrained AMS infrastructure. Integrated AMS interventions combining institutional antibiograms and their reinforcement, audit and feedback mechanisms, establishment of clear AMS structures and laboratory capacity strengthening are urgently needed to mitigate AMR and optimize surgical antibiotic use.

## Introduction

Antimicrobial resistance (AMR) is a growing global public health challenge, disproportionately affecting low- and middle-income countries (LMICs). In Africa, where the burden of surgical site infections is over 40%, AMR is estimated to contribute to at least one million deaths per annum [1–3]. In Uganda, hospital surveillance data shows resistance as high as 44% to 100% for many first line antibiotics, contributing to treatment failure, prolonged hospitalization and higher morbidity and mortality [4,5]. Surgical wards represent high-risk settings for AMR, due to high antibiotic exposure for perioperative surgical antibiotic prophylaxis (SAP) and empirical therapy, often in contexts of limited microbiological guidance and inadequate infection prevention and control (IPC) practices [6,7].

Despite recognition of the AMR burden, there is limited context-specific evidence on the prevalence, patterns, and drivers of antibiotic use in adult surgical wards in Uganda. Most studies to date focus on hospital-wide or national-level estimates, providing minimal insight into ward-level prescribing behaviours or the clinical and contextual factors that influence them. The perspectives, attitudes, and decision-making processes of HCWs regarding antibiotic use remain poorly characterized. Understanding these qualitative dimensions is essential because prescribing behaviour is shaped not only by clinical guidelines but also by experiential, social, and systemic factors within the hospital setting [8]. Without integrating qualitative insights, efforts to understand AMR risk factors in surgical wards remain incomplete.

This study aimed to address these knowledge gaps by: (i) Describing the patterns of inpatient antibiotic use, (ii) Characterizing antimicrobial susceptibility profiles of common bacterial pathogens isolated from surgical patients, and (iii) Exploring HCWs perceptions, experiences, and decision-making processes regarding antibiotic prescribing. By combining quantitative point prevalence and microbiological data with qualitative insights from frontline providers, we sought to generate a comprehensive understanding of the drivers of antibiotic use and AMR in surgical wards, providing critical evidence for policy and practice in Ugandan tertiary hospitals.

## Methods

### Study design and setting

A cross sectional convergent mixed-methods study was conducted between September and November 2025, on the adult surgical wards of MNRH in Kampala, Uganda. It consisted of a point prevalence survey of inpatient antibiotic use (23^rd^ Sep-3^rd^ October 2025) and a retrospective analysis of microbiology data for patients admitted to MNRH surgical wards from June 2023 to August 2025. Qualitative IDIs (23^rd^ Sep to 30^th^ Nov 2025) were also conducted, exploring healthcare worker (HCWs’) perceptions and lived experiences on AMR, antibiotic prescriptions and antimicrobial stewardship practices. At the time of this study, antibiotic prescriptions both pre- and post-operatively were based on clinician discretion, antibiotics available in hospital pharmacy and the 2023 Uganda Clinical Guidelines (UCG) [9].

### Study participants

The point prevalence survey targeted adult surgical patients present on the ward by 8 am on the morning of the survey and received an antibiotic within the last 24 hours. The microbiological data analysis focused on results of specimens collected from the surgical wards and processed at the MNRH Microbiology Laboratory or the Makerere University Clinical Microbiology Laboratory (MUCML) between June 2023 and August 2025. Results that had critical or high priority pathogens including; *Acinetobacter baumannii, Pseudomonas aeruginosa, Staphylococcus aureus, Escherichia coli, Proteus mirabilis, Klebsiella pneumoniae* and *Citrobacter freundii* were extracted [10].

The IDIs focused on HCWs in the surgical wards including; Surgical specialists, Resident doctors, Medical Officers, Pharmacists or Nurses. HCWs had to have worked on the ward for at least one month by the time of study start and were present on the ward at least 3 days each week to actively engage in prescription, dispensing or drug administration while on duty. We excluded HCWs who were unable to be physically present for the interviews.

### Study procedures

#### Point prevalence surveys on inpatient antibiotic use.

The point prevalence survey was conducted by trained healthcare workers using an adapted WHO Point Prevalence Survey (WHO-PPS) tool (S1 File), which had been pre-tested on 10 files from a non-surgical ward and digitized. A survey schedule was developed, and PPS was conducted sequentially across all surgical wards (S1 Fig). On each survey day, the inpatient register was reviewed to identify patients who had received antibiotics in the previous 24 hours, and eligible records were line-listed by the study team. Medical records were then reviewed consecutively in ascending inpatient number order until data from all eligible patient records was abstracted. Data abstracted included patient demographics, antimicrobial resistance risk factors, clinical diagnoses, antibiotic indications, prescriptions, dose, route and duration of regimen, using information from patient files, medication charts, clinician and nursing notes, and laboratory reports. All data were anonymized using unique study identifiers, with no patient names recorded. Clarification was sought from ward clinicians where necessary, without direct patient interaction. Data were uploaded into a secure password-protected electronic database and reviewed for completeness and accuracy by the study team.

### AMR survey

Microbiological data (sample type, isolate identified and susceptibility profiles) was extracted along with patient identification, ward, age and sex from the electronic laboratory information systems of MNRH Microbiology and MUCML laboratories and captured it onto the WHO PPS microbiology data form. Both laboratories utilized automated incubation and monitoring for blood cultures using BD BACTEC FX40 system (Becton Dickinson and Company, Sparks, MD, USA). For positive blood cultures and majority other specimens collected, they used incubation on solid media, microscopy and biochemical techniques for isolate identification and then antimicrobial susceptibility testing using the Kirby Bauer disc diffusion method according to the Clinical and Laboratory Standards Institute (CLSI) guidelines 2023–2025 [11–13]. Both laboratories also utilized identical laboratory standard operating procedures for culture and sensitivity, handling contaminants, and utilized known American Type Culture Collection (ATCC) controls among other quality control techniques in the culture and sensitivity process. Records of patients aged 18 + , who had been admitted to the surgical wards were included. The extracted data was entered into Microsoft Excel and cleaned. Missing data entries were cross-checked from the corresponding laboratory workbooks and entered into a Microsoft Excel sheet.

### HCWs IDIs

An open-ended interview guide with probes developed by the authors, was used to guide the IDIs using key thematic questions relevant to the study objectives. It also consisted of prompts designed to explore participants’ perceptions and experiences. The interview guide was pretested on five participants similar to the study population. Eligible participants were approached in-person and provided with detailed information about the study and informed consent forms. Participants were interviewed by a female study staff (DA (Nurse) in a quiet study room, at their convenient time for 45–60 minutes. Interviewing study staff had undergone prior training on qualitative methodology and data collection and didn’t have any relationship with participants or interest in research topic. All interviews were audio recorded securely stored on password-protected devices. Notes were used to document major themes arising during the interviews. Interviews were transcribed verbatim (FMM, DA) in English language and cross-checked by the principal investigator and qualitative specialist (FMM, AT) for accuracy.

### Statistical methods

#### Sample size and sampling technique.

The sample size for the point prevalence survey was calculated using the single population proportion formula assuming a 95% confidence level, 5% margin of error, and an estimated prevalence of antibiotic use of 50% based on previous literature [14]. The initial sample size obtained was 323 participants. Because the source population was below 10,000, finite population correction was applied using an estimated eligible population of 190 patients, yielding a final minimum sample size of 127 patient records. We used consecutive sampling of all eligible participants’ records to obtain PPS and AMR records for review and data abstraction. All eligible records were included and abstracted.

The sample size for the IDIs was estimated at 30 HCWs as per existing literature on recommended sample sizes for qualitative studies [15], including at least two from each of the following categories: Resident doctors, pharmacists, nurses, medical officer and surgical specialists. We purposively selected enrolled study participants until we achieved saturation. Thematic saturation was defined as the point in data collection, at which no new or significant themes or patterns were reported by the participants. This was evaluated through post interview debrief forms in which any new emerging themes for each interview were documented.

### Point Prevalence Survey (PPS) data analysis

Antibiotic use patterns constituted the primary unit of analysis and were linked to individual patients. Patient-level characteristics were summarized using frequencies and percentages for categorical variables, and medians with interquartile ranges (IQR) for continuous variables. The prevalence of antibiotic use was defined as the proportion of surveyed patients receiving at least one systemic antibacterial agent on the day of the survey. Antibiotic use patterns were described overall, and key indicators included the median number of antibiotics prescribed per patient and the distribution of prescriptions according to the WHO Access, Watch, and Reserve (AWaRe) classification. Antibiotic classes and individual agents were summarized using frequencies and percentages. Prescribing quality indicators recommended in the WHO PPS protocol were assessed, including compliance with Uganda Clinical Guidelines 2023 (UCG), and the duration of SAP. Guideline compliance was defined and calculated as the proportion of antibiotic agent choices made as indicated in the UCG (antibiotic choice only). All PPS indicators were presented overall and stratified by prescriber cadre, and AWaRe category where appropriate. All statistical analyses were conducted using R version 4.2.2 and STATA 19.

### Antimicrobial Resistance (AMR) data analysis

Antimicrobial resistance (AMR) data was summarized as the percentage of each species of organisms that were non-susceptible (classified as resistant or intermediate on susceptibility testing) to each test antibiotic as per CLSI guidelines [16]. Non-susceptibility patterns were presented graphically to highlight antimicrobial agents with high prevalence of non-susceptibility. All statistical analyses were conducted using R version 4.2.2 and STATA 19. To prevent data inflation from repeat sampling, a strict de-duplication protocol was applied: only the first isolate per patient per unique bacterial species within the same clinical episode was included. Duplicate isolates of the identical species from the same patient were excluded. Organism-antimicrobial combinations with intrinsic resistance or without established interpretive criteria were either excluded from analysis or reported as non-recommended (NR) in accordance with CLSI guidelines.

### Qualitative data analysis

Thematic content analysis was conducted as per the six-phase framework by Braun and Clarke (2006) [17]. After transcribing the audio recordings and typing them in word documents, we (FMM, DA, AT) conducted a thematic analysis using a systematic, multi-step approach to ensure depth and rigor. First, immersive reading of all interview transcripts was utilized, to become thoroughly familiar with the data, carefully marking significant statements and writing analytic memos to capture emerging ideas and reflections. In the second step, line-by-line open coding was performed (FMM, AT) on 25% of the selected transcripts to inductively generate an initial set of codes. These preliminary codes were developed manually on paper and used to construct a coding framework that guided subsequent analysis. During the second cycle of coding, all transcripts were imported into NVivo version 12 to facilitate systematic data organization and retrieval. The codes were then reviewed, compared, and grouped into broader categories based on patterns, similarities, and conceptual linkages, leading to the development of overarching themes. Finally, representative quotations corresponding to each theme were identified, extracted, and organized to support and illustrate the findings presented in the results narrative.

### Study variables

The primary PPS outcome was prevalence and patterns of antibiotic use. Prevalence of antibiotic use was defined as the proportion of patients receiving at least one systemic antibacterial agent within the last 24 hours. The unit of analysis was the antibiotic prescription, linked to individual patients. Key variables included ward-specific antibiotic prevalence, median number of antibiotics per patient, antibiotic class, individual agent, route and indication (therapeutic or prophylactic). Antibiotics were categorized using the WHO AWaRe classification. Prescribing quality indicators included compliance with UCG, proportion of SAP, duration of prophylaxis (≤24 hours versus >24 hours) and cadre of prescribing HCW.

The primary AMR survey outcome was non-susceptibility, defined as isolates classified as intermediate or resistant to each antibiotic according to CLSI criteria. Each laboratory-confirmed isolate represented a unique observation. Variables included specimen type, organism identified, antimicrobial agents tested, and susceptibility results. Non-susceptible proportions were calculated for each organism-antibiotic combination as the proportion of non-susceptible isolates among those tested, in line with WHO GLASS standards.

### Integration of PPS, AMR and IDI results

Integration of quantitative and qualitative findings was performed using a convergent mixed-methods approach. Quantitative (PPS and AMR surveys) and qualitative IDIs data was collected and analysed independently and then brought together for interpretation. Findings were compared, contrasted and triangulated to assess areas of convergence, complementarity and divergence. Quantitative PPS patterns were examined alongside qualitative explanations to understand underlying drivers of prescribing behaviour. Patterns of antibiotic use were interpreted considering AMR data to determine whether prescribing aligned with local susceptibility profiles. Where discrepancies arose, qualitative findings were used to explore contextual and systemic explanations. A joint display matrix was developed to align key quantitative indicators with corresponding qualitative themes.

### Ethical considerations

This study was conducted according to the Declaration of Helsinki, Good Clinical Practice Guidelines and guidelines by the Uganda National Council of Science and Technology. Ethical approval was sought from the Research and Ethics Committee of Makerere University School of Public Health (SPH-2025–899) and administrative clearance was obtained from MNRH in Uganda (MHREC 2967). This study was registered with the Uganda National Council of Science and Technology (HS6321ES). A waiver of informed consent was obtained for the cross-sectional aspect of the study as all procedures involved data abstraction from patient files and there was no interaction with patients whatsoever. For the qualitative aspect, only HCWs who provided voluntary written informed consent were enrolled on the study. HCWs enrolled on the study received a time compensation of USD 15 after each interview.

## Results

### PPS results

#### Characteristics of study population.

Among the 303 surgical inpatients present on the wards during PPS, 193/303 (63.7%) (95% CI: 58.0–69.12) had at least one antibiotic prescribed and were included in the analysis, of these, 125/193 (64.8%) were male (Table 1). The median age was 40 years (IQR 27–58) with 106/193 (54.9%) being 18–44 years of age. A total of 135/193 (70.0%) had undergone surgery since current admission, 133/193 (68.9%) had peripheral vascular catheter *in situ* and 62/193 (32.1%) had comorbidities.

**Table 1:** Sociodemographic and clinical characteristics of the PPS participants.

Characteristic (N = 193)	Frequency n (%)
**Median Age in years (IQR)**	40 (27-58)
**Sex**	
Female	68 (35.2)
Male	125 (64.8)
**Had surgery since admission**	
No	54 (28.0)
Yes	135 (70.0)
Unknown	4 (2.1)
**Central Vascular Catheter**	
No	182 (94.3)
Yes	7 (3.6)
Unknown	4 (2.1)
**Peripheral Vascular Catheter**	
No	54 (28.9)
Yes	133 (68.9)
Unknown	6(3.1)
**Urinary Catheter**	
No	140 (72.5)
Yes	43 (22.3)
Unknown	10(5.2)
**Intubation**	
No	189 (97.9)
Yes	4 (2.1)
**Comorbidities¶**	
No	131 (67.9)
Yes	62 (32.1)
**Transferred from lower hospital**	
No	149 (77.2)
Yes	31 (16.1)
Unknown	13 (6.7)
**Hospitalized in the past 90 days before this admission**	
No	6 (3.1)
Yes	183 (94.8)
Unknown	4 (2.1)
**Ward**	
General and Gastrointestinal surgery	33 (17.1)
Orthopaedic and Trauma surgery	40 (20.7)
Neurosurgical and Spine surgery	59 (30.6)
Burns, Plastics and Reconstructive surgery	25 (13.0)
Cardiothoracic and other specialized surgery	36 (18.7)

¶-Commonest comorbidities included: Anaemia excluding sickle-cell (n = 8, 12.9%), Cancer/malignancy (n = 3, 4.8%), Diabetes mellitus (n = 10, 16.2%), HIV disease and Tuberculosis (n = 10, 16.2%), Hypertension (n = 15, 24.2%) and substance use disorder-alcohol (n = 4, 1.6%).

### Prevalence of antibiotic use

**Fig 1:**
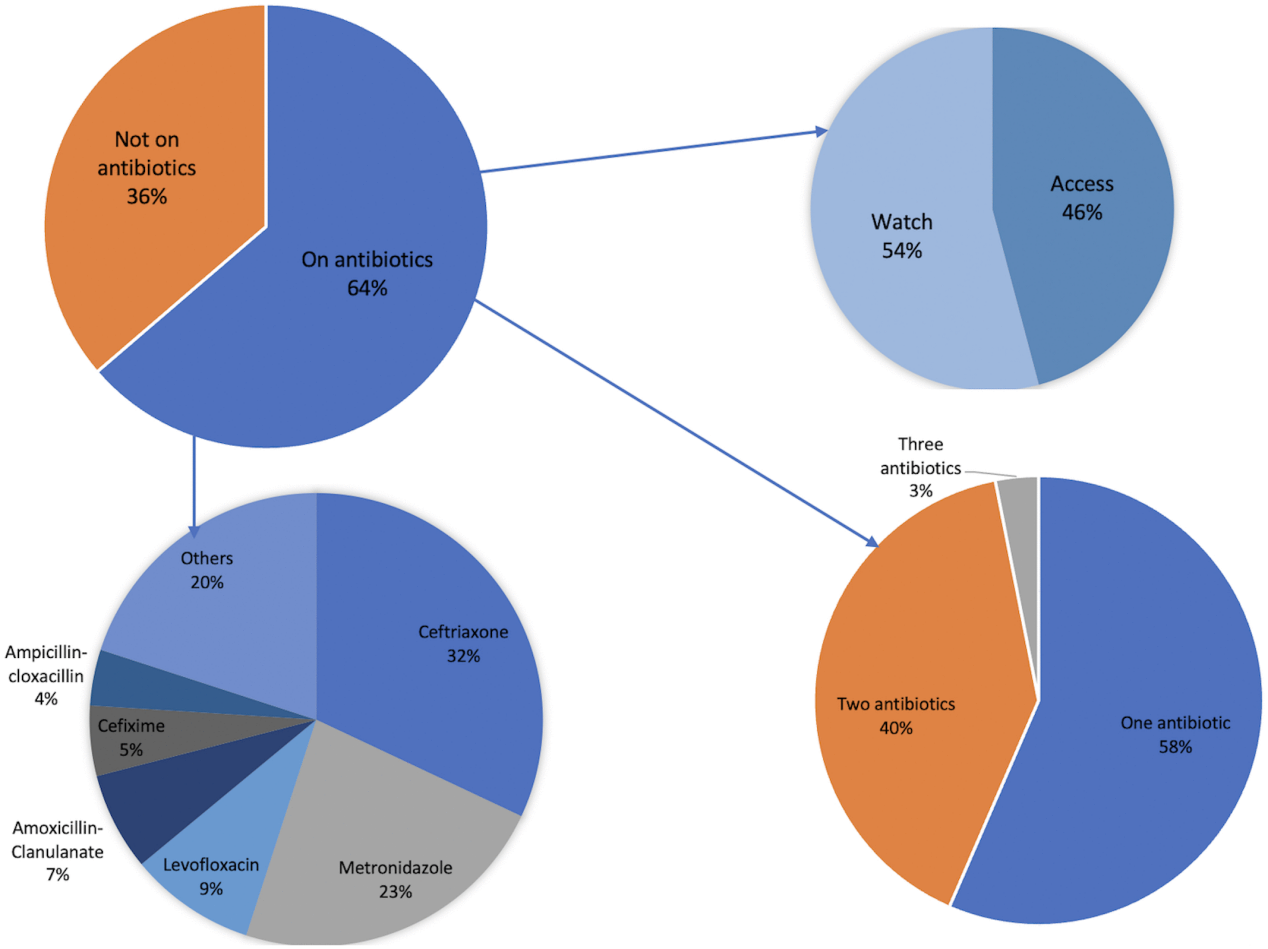
Prevalence of antibiotic use, AWaRe and antibiotic agents used.

The median number of antibiotics prescribed per patient was one (IQR:1–2) with 109/193 (56.5%) patients being on one antibiotic and 78/193 (40.4%) on two antibiotics. Of all the prescriptions reviewed, only 136/281 (48.4%) were compliant to UCG (Fig 1).

### Antibiotics prescribed

281 antibiotics were prescribed in total to 193 patients, with 89/281 (31.7%) (95% CI: 26.27–37.46) being ceftriaxone, 66/281 (23.5%) (95% CI: 18.66–28.89) metronidazole and 24/281 (8.5%) (95% CI: 11.61–20.44) being levofloxacin (Fig 1). Of all prescriptions, 152/281 (54.1%) (95% CI: 48.08–60.08) were in the Watch category and 129/281 (45.9%) (95% CI: 39.9–51.9) were in Access category. Antibiotics were prescribed for SAP 256/281 (91.1%), with 239/256 (93.4%) of these SAP antibiotics given as multiple doses beyond first 24 hours (Table 2). Prescribing was done by Resident doctors 120/281 (42.7%) and Surgical specialists 79/281 (28.1%). Directed antibiotic therapy was at 10/281 (3.6%).

**Table 2:** Summary of antibiotic use characteristics.

Characteristics (N = 281)	Frequency n (%)
**Indication type**	
Community-acquired infection	10 (3.6)
Hospital-associated infection	3 (1.1)
Medical prophylaxis	10 (3.6)
SAP: surgical antibiotic prophylaxis	256 (91.1)
Other	2(0.7)
**SAP duration (n = 256)**	
One dose	15 (5.9)
Multiple doses within first 24 hours	2 (0.8)
Multiple doses beyond first 24 hours	239 (93.4)
**AWaRe Classification**	
Access	129 (45.9)
Watch	152 (54.1)
**Prescriber type**	
Clinical Officer	5 (1.8)
Junior house officer	56 (19.9)
Medical Officer	21 (7.5)
Resident doctors	120 (42.7)
Surgical Specialist	79 (28.1)

### AMR results

Among the 1,879 microbiology tests conducted between June 2023 and August 2025, 480/1879 (25.5%) were conducted on adult surgical inpatients and were included in this study (S1 Table). The median age of the participants was 38 years (IQR: 28–53.5), with 304/480 (63.3%) being 18–44 years and male 329/480 (68.5%). The sample types processed for culture and sensitivity testing included pus 335/480 (69.8%) and urine 36/480 (7.5%) among others (S2 Fig).

### Bacterial pathogens Isolated on culture

**Fig 2:**
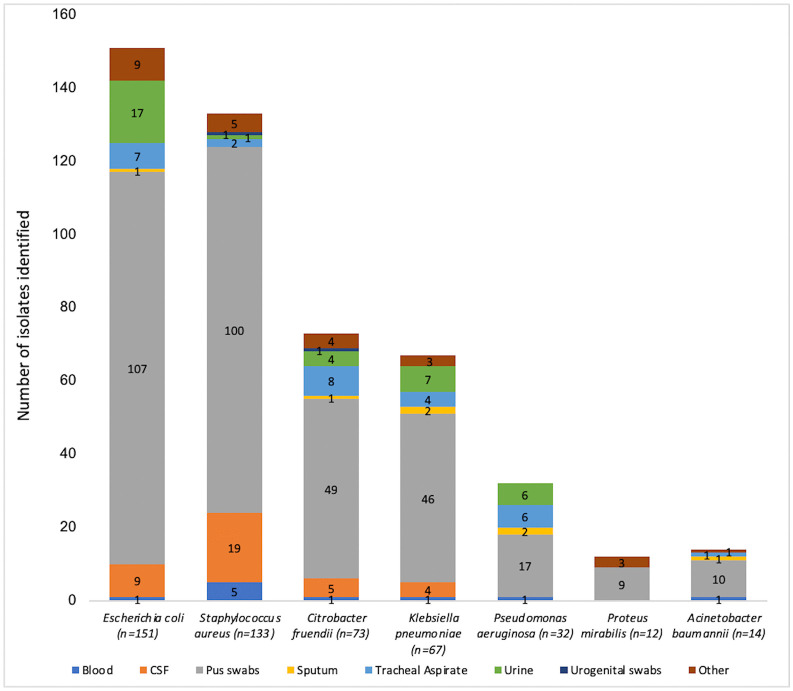
Bacterial pathogens isolated on culture across sample types (N = 482).

Of the 482 bacterial pathogens isolated from 480 specimens, 151/482 (31.3%) (95% CI: 27.35–35.60) was *Escherichia coli*, followed by *Staphylococcus aureus* at 133/482 (27.6%) (95% CI: 23.79–31.75), *Citrobacter freundii* at 73/482 (15.1%) (95% CI: 12.22–18.62) and *Klebsiella pneumoniae* at 67/482 (13.9%) (95% CI: 11.10–17.28) (Fig 2).

*Other = other sample types include: ascitic, pleural, joint and endocardial fluid aspirations.

### Drug susceptibility profiles of isolated bacterial pathogens

Non-susceptibility to individual antibiotics was observed across all isolates. *Escherichia coli* showed 142/151 (94.0%) non-susceptibility to Ceftriaxone, 124/151 (82.1%) to Cefuroxime and Ciprofloxacin, 113/151 (74.8%) to Cefepime, 81/151 (53.6%) to Amoxicillin-Clavulanic acid, 32/151 (21.2%) to Imipenem and 9/151 (6.0%) to Meropenem (Table 3). *Citrobacter freundii* showed 66/73 (90.4%) non-susceptibility to cefuroxime, 63/73 (86.3%) to Ceftriaxone, 61/73 (83.6%) to Ciprofloxacin and 22/73 (30.1%) to Imipenem. *Klebsiella pneumoniae* showed 63/67 (94.0%) non-susceptibility to Cefepime, 61/67 (91.0%) to Cefuroxime, 59/67 (88.1%) to Ceftriaxone, 53/67 (79.1%) to Ciprofloxacin, 41/67 (61.2%) to Amoxicillin-clavulanic acid, 25/67 (37.3%) to Imipenem and 15/67 (22.4%) to Meropenem. *Staphylococcus aureus* showed 44/133 (33.1%) non-susceptibility to Cefoxitin, 109/133 (82.0%) to Ciprofloxacin, 71/133 (53.4%) to Gentamicin and 77/133 (57.9%) to chloramphenicol.

**Table 3:** Antibiogram showing percentage susceptibility across bacterial pathogens (%S=(S)/(I + R + S)*100).

Organism (n = number of isolates)	Amoxicillin/ clavulanic acid	Ampicillin	Azithromycin	Cefepime	Cefoxitin	Ceftriaxone	Cefuroxime	Chloramphenicol	Ciprofloxacin	Erythromycin	Gentamicin	Imipenem	Meropenem	Tetracycline	Sulfamethoxazole/ Trimethroprim
*Escherichia coli* (n = 151)	46	7	NR	25	—	6	18	56	18	NR	42	79	94	67	16
*Citrobacter freundii* (n = 73)	50	IR	NR	14	—	14	10	44	16	NR	61	71	75	25	8
*Klebsiella pneumoniae* (n = 67)	39	IR	NR	6	NR	12	9	46	21	NR	53	63	78	—	22
*Pseudomonas aeruginosa* (n = 32)	IR	—	NR	75	—	IR	IR	IR	—	NR	61	85	71	—	IR
*Proteus mirabilis* (n = 12)	100	20	NR	—	—	36	—	40	63	NR	75	75	—	IR†	—
*Acinetobacter baumannii* (n = 14)	—	—	NR	—	—	—	—	—	—	NR	—	—	20	—	—
*Staphylococcus aureus* (n = 133)	60	NR	50	—	36‡	—	60	69	35	19	51	—	—	54	44

— = Not tested/not reported, NR = Not recommended for routine interpretation or reporting due to lack of established clinical breakpoints or poor clinical utility for the organism group, IR = Intrinsic resistance; organism is naturally resistant to the antimicrobial agent and results should not be clinically interpreted, † Proteus spp. commonly demonstrate intrinsic reduced susceptibility to tetracyclines, ‡ Cefoxitin susceptibility in Staphylococcus aureus was used as a surrogate marker for methicillin susceptibility (MRSA screening) according to CLSI guidelines.

### Qualitative results

We screened 49 HCWs and enrolled 29 including: 3 specialist surgeons, 12 resident doctors, 2 pharmacists and 12 nurses into this arm of the study (S2 Table). Eighteen were male and median duration in service was 4.0 years (IQR: 2.5–5.0). 20 eligible HCWs declined participation in the study as they couldn’t afford a one-hour sit-down for the IDIs. All IDIs were conducted once without repetition. Three major themes arose from the qualitative data analysis: 1) Determinants of antibiotic use, 2) Perceptions on AMR and 3) perceptions on AMS.

### Determinants of antibiotic use

Across IDIs, several respondents (10/29) described prescription practice as largely driven by experience, training, and drug availability rather than standardized protocols. Many respondents explicitly stated that no formal, written, ward specific antibiotic use protocols existed, even where national guidance was acknowledged, implementation was uncertain. In the absence of local standard operating procedures (SOPs), prescribing relied on inherited norms based on school experience and personal knowledge:

*“...I wouldn’t say there’s a written guideline… It’s not there”.* IDI-Lgit-029. *“They are the Uganda clinical guidelines… I really don’t know if we really go in so much to follow the step-by-step prescription they are suggesting”.* IDI-Thor-001. *“...We use what we know works based on experience and knowledge that has been passed down to us”.* IDI-Lgit-029

Clinicians relied on clinical judgment and experience to escalate to broad-spectrum antibiotics for last resort antibiotics like meropenem or vancomycin when severe infections or high-risk patients were encountered, even if this departed from UCG**.**

*“Sometimes you find the patient is having a very bad infection… you start with an antibiotic which is high in the ladder because you think that if you don’t start it right early, we may lose the patient. Even some patients, we start… meropenem or vancomycin.”* IDI-Neur-018

Broad-spectrum antibiotics, particularly ceftriaxone, metronidazole, meropenem, and levofloxacin were reported by majority respondents (24/29) as frequently used for both prophylactic and therapeutic purposes in the adult surgical wards of MNRH, grounded in “*we know what works, based on experience and knowledge that has been passed down to us”* IDI-Lgit-029. These were chosen because of their availability, affordability, proven effectiveness, and ease of administration.

*“The most commonly prescribed are cephalosporins, that is, ceftriaxone, cefixime, and then metronidazole. We also prescribe ciprofloxacin and levofloxacin, mainly* *because those are the most available, and at times, those are the ones that the bacteria respond to”.* IDI-Ugit-028

Ward environment lapses like overcrowded patient areas, unhygienic conditions and past experiences with infections drove clinicians to use antibiotics more broadly and pre-emptively, even when UCG suggested that single prophylactic use would suffice. Prophylaxis was framed as standard surgical principle and justified by anticipated contamination.

*“...in our setting, we have a fear that the patient’s hygiene is poor, the environment where we work is not very safe, sometimes we work in theatres that are very hot, people are sweating, because we fear that the risk of wound infection is high. We tend to always put the patients on antibiotics when actually you feel that in an ideal situation, they don’t need antibiotics… for fear that in case there’s a contamination.”* IDI-SHO-Orth-013

Delays in conducting culture and sensitivity sometimes up to two weeks further complicated timely directed therapy and prior antibiotic exposure from previous hospital admissions often rendered commonly used antibiotics to be perceived by majority clinicians (26/29) as ineffective.

*“Actually, on that ward, they normally don’t perform culture and sensitivity, it’s common. So, you find a patient, these patients are always in hospital for three months and above, they usually get hospital acquired infections, so you give the patient an antibiotic for a week, seven days later, no change and the patient is deteriorating. That’s when they decide to do a culture.”* IDI-Throc-003

Availability of free services also shaped prescription choices. Clinicians stated that even when tests like cultures and drugs were *“...entirely free of charge,”* IDI-Phar-Neur-027, the supply was inconsistent and the cost of antibiotics thus limited optimal prescribing. The price of antibiotics influenced which drugs were chosen, sometimes favouring cheaper but less ideal options.

### Perceptions on AMR

Across IDIs, several respondents (26/29) narrated antimicrobial resistance (AMR) as a persistent and escalating clinical challenge marked by treatment failure, limited antibiotic options, financial strain, and difficult prescribing decisions. Respondents described repeated antibiotic failures. Ineffectiveness of antibiotics due to resistance was frequently associated with prior antibiotic use. Respondents described prolonged empirical treatment before culture guidance. Some (6/29) HCWs narrated that patients reportedly arrived after multiple prior antibiotic exposures: *“They come… when they have already had a cocktail of antibiotics”* IDI-NO-Urol-010. Multidrug resistance among elderly patients was described as severe: *“All drugs are almost ineffective”* IDI-SHO-HDU-017. AMR was also perceived by many HCWs (19/29) to result in delayed recovery and stunted wound healing. Clinically, resistance led to treatment failure, prolonged infections, complications, and preventable deaths. Respondents recounted cases where patients failed to respond to multiple antibiotics and died. Resistant infections often forced clinicians to escalate to costly “big gun” antibiotics, sometimes without culture results.

*“...Sometimes you find we have gone for some big antibiotic… you choose to save the patient… but you are risking the future*.” IDI-Neur-018

HCWs also acknowledged that hospital-acquired infections and poor adherence to infection prevention and control (IPC) practices, such as inadequate hand hygiene or improper glove use, facilitated the spread of resistant organisms (*super bugs*) from patient to patient.

*“If I interact with a patient with a bug and I don’t do proper hand washing… transfer the infection… there’s an emergence of more resistant bugs due to spread from patient to patient.”* IDI-SHO-Trau-007

### Perceptions on AMS

Several respondents (28/29) narrated basic awareness of core AMS practices, particularly rational prescribing, correct dosing, culture-guided therapy, and avoidance of unnecessary antibiotics. However, understanding was often individualized and experiential rather than institutionalised. Across Respondents, antimicrobial stewardship (AMS) was generally understood as the responsible, rational, and evidence-based use of antibiotics to prevent resistance, though the depth and clarity of understanding varied. Many respondents described stewardship in terms of vigilance and responsibility. One consultant noted it involves;

*“...being keen and observant and responsible to ensure you prevent resistance… avoid unnecessary prescription… monitor… do culture and sensitivity”* IDI-Cons-Lgit-029.

While some Respondents acknowledged the presence of a hospital-based formal antimicrobial stewardship (AMS) subcommittee, awareness, orientation, and operational clarity varied across cadres. Formal stewardship structures were perceived as limited or inactive. At consultant level, there was recognition of a formal stewardship presence within the hospital; at management level, but not on the ward. One consultant acknowledged a hospital-level team, but noted, *“in the unit we don’t have a specific team.”* IDI-Cons-Lgit-029 and another reported, *“...I have not been oriented to one [AMS]...”*. IDI-NO-LGIT-014. One respondent also stated, *“I know it is there”*, and described its expected role.

*“...being at the forefront of determining what bugs are available… monitoring the organisms… championing standard operating procedures… [and] giving feedback to all the teams.”* IDI-CONS-UGIT-026.

### Integration of quantitative and qualitative results

Quantitative results showed a prevalence of antibiotic use, of antibiotics in the Watch (54.1%) category compared to the Access category (45.9%), with Ceftriaxone (31.7%), being the most prescribed. SAP was given using these antibiotics, as multiple doses beyond first 24 hours (93.4%). The commonest bacterial pathogens isolated from among surgical patients were *Escherichia coli* (31.3%) and *Staphylococcus aureus* (27.6%), which pathogens had 80–100% non-susceptibility to Ceftriaxone.

Qualitative data explained the factors affecting prescription patterns: there was no ward SOP on antibiotic prescription, the hospital environment was overcrowded and clinicians perceived IPC practices to be inadequate. Rational antibiotic use was mainly determined by availability of drugs, cost and turnaround time of microbiology tests and drugs, lack of structured, documented guidance on antibiotic choice and lack of knowledge on AMR and proper AMS practices. Some clinicians understood AMR and AMS but they were not aware of the existence of the hospital level antimicrobial stewardship subcommittee or its roles. There were no ward specific personnel for AMS (S3 Table).

## Discussion

This study aimed to describe inpatient antibiotic use and AMR patterns as well as explore drivers of antibiotic use on the surgical wards of MNRH. We found a high prevalence of antibiotic use, with nearly two thirds of patients on antibiotics, predominantly for SAP. Most patients were on prolonged multi-day SAP regimens contrasting global and national recommended durations. Compliance to UCG, the only known national standard on the unit, was less than 50%. Watch antibiotics (54.1%) were the most used instead of Access antibiotics (45.9%), resulting in an inverted picture compared to the WHO AWaRe framework which recommends at least 70% Access antibiotics usage [18]. Ceftriaxone, Metronidazole and Levofloxacin were the most prescribed agents. Multiple critical and high priority pathogens had been isolated on culture and sensitivity among surgical patients over the last two years, these pathogens had high non-susceptibility to cephalosporins, fluoroquinolones and B-lactam/B-lactamase combinations but majority were susceptible to Carbapenems. Qualitative data reinforced these findings; HCWs narrated marginal awareness of AMR, AMS and AMS subcommittee presence in MNRH. Details of formal AMS subcommittee structures were unknown, ward specific AMS structures were absent, multiple health system constraints and clinician perceptions contributed to the gap between AMS/AMR knowledge and prescribing practice.

We observed suboptimal compliance to national clinical guidelines and WHO AWaRe framework, with over 54% use of Watch antibiotics and less than 46% for Access antibiotics. The commonest agents used were Cephalosporins followed by fluoroquinolones and B-lactam/B-lactamase combinations. Third and fourth generation Cephalosporin non-susceptible *Escherichia coli*, *Citrobacter freundii, Klebsiella pneumoniae* and *Pseudomonas aeruginosa* as well as methicillin resistant *Staphylococcus aureus* were the commonest isolated pathogens from surgical patients, identical to resistance trends in MNRH over the last 15 years [7,19,4]. The high prevalence use of higher generation Cephalosporins for SAP, particularly Ceftriaxone, is a common practice in Africa, widely documented for their affordability, availability and inclusion on essential medicines list [20]. These findings reflect a tilt towards unrecommended use of broader spectrum agents in empirical therapy. This practice was explained in IDIs as a common practice due to distrust in IPC practices, perceived high risk of surgical site contamination and uncertainty in effectiveness of Access agents in patients with recent antibiotic exposure, also narrated in similar studies [21].

The lack of hospital specific microbiology surveillance, data use and absent antibiotic prescription SOPs further encourages this practice by fostering uncorroborated perceptions on antibiotic effectiveness in this setting. Cephalosporins mostly target skin and gut flora, hence being excellent SAP agents for majority surgical procedures, however, the extensive use of these agents in MNRH, for prolonged SAP, without consideration of AMR patterns from hospital data could be a matter of concern. Ceftriaxone SAP, despite its effectiveness may not be an appropriate uniform regimen for all surgical inpatients. A more impactful approach would be the stratification of patients according to risk of multi-drug resistant or severe infections and subsequent use of individualised SAP regimens for the high-risk group [22]. While lower-risk patients can successfully utilize standard first- or second-generation cephalosporins, high risk patients could receive tailored, optimised higher generation antibiotic agents. Guarding these restricted agents is increasingly critical given emerging global reports of complex co-resistance mechanisms, which threaten efficacy of our remaining last line therapies [23].

The predominance of Watch antibiotics warrants attention, considering global efforts to rebalance antibiotic consumption toward Access agents. The WHO AWaRe framework emphasized increasing the proportion of Access consumption to at least 60% consumption of total consumption to mitigate resistance selection pressure [24]. In our study, Watch antibiotics accounted for more than half of prescriptions, indicating a prescribing profile that may contribute to sustained selection for ESBL-producing organisms and other multidrug resistant organisms. Qualitative findings suggest that this practice is not completely preference driven but rather reflects risk-averse clinical reasoning in an environment characterized by prior antibiotic exposure and uncertainty regarding local susceptibility patterns. The qualitative data further contextualizes this practice, as documented resistance trends and reports of treatment failure may reinforce clinician concerns about first line agent effectiveness. This inverted AWaRe framework finding underscores the importance of coupling antibiotic consumption surveillance with robust, timely antibiogram dissemination and utilization. Without ward specific AMR data feedback, empirical escalation may become entrenched, normalizing Watch and Reserve agents use as default, rather than exceptional practice.

The discrepancy between AMS awareness and prescribing behaviour represents a very important insight of this study. HCWs demonstrated basic understanding of stewardship principles, including rational prescribing, culture-guided therapy, and avoidance of unnecessary antibiotics. However, quantitative indicators revealed limited compliance with UCG, prolonged SAP, and minimal reliance on culture in antibiotic choice. This gap is mediated by systemic constraints rather than knowledge deficits alone. Long laboratory turnaround times, drug and laboratory reagent availability inconsistencies and staffing shortages collectively shape prescribing realities. Additionally, the absence of visible ward-level antibiotic prescription SOPs, SAP guidelines and structured prescription audit-feedback mechanisms limits accountability and reinforcement of best AMS practices. These findings align with studies that emphasize the role of structural and contextual determinants in shaping prescribing behaviour [25]. Importantly, this demonstrates how AMS in this setting is largely informal and hierarchical, relying on professional norms rather than institutionalized monitoring systems. There is a need for qualitative studies to explore what drives clinicians to over prescribe watch antibiotics. Strengthening stewardship therefore requires not only training, but also systems redesign, including laboratory strengthening, standardized documentation of context specific antibiotics and SAP guidance, protected AMS time, and regular feedback loops linking resistance data to prescribing units.

The pattern and high prevalence of antibiotic use observed in this study is consistent with other studies from LMIC settings [26]. Previous studies have highlighted 50–80% antibiotic prevalence among inpatients, with surgical wards often having higher rates [27]. The prolonged SAP, which extended beyond the first 24 hours in majority cases, is a notable finding which contrasts with evidence-based guidelines that recommend a single dose regimen within 24 hours for most clean and clean-contaminated procedures and exceeds that reported in comparable PPS studies [28]. Integrated with qualitative themes suggesting high infection risk, mistrust in IPC practices and overcrowded hospital environments as explanations for prolonged prophylaxis, local contextual drivers may be exerting a stronger influence on prescribing behaviour than national guidelines.

Several strengths and limitations merit consideration. A major strength of this study is its convergent mixed-methods design, which enabled triangulation of antibiotic use data, microbiological data, and contextual explanations from frontline HCWs, providing a nuanced understanding of antibiotic use in surgical populations. However, limitations include the cross-sectional nature of the PPS, which captures prescribing patterns at a specific time point and may not fully reflect temporal variability. AMR findings may be subject to selection bias and are to be interpreted cautiously as cultures are more likely to be obtained from severe or complicated cases, which could have overestimated resistance prevalence and patterns compared to a general surgical ward population. Additionally, qualitative findings may be subject to social desirability bias, particularly when discussing AMS awareness. Lastly, this was a single centre study conducted on surgical wards of MNRH, which limits generalizability of the findings to other health facilities in Uganda.

In summary, this study demonstrates that prolonged SAP, and predominant Watch antibiotic prescribing, are occurring simultaneously within a context of documented AMR and constrained AMS infrastructure. While HCWs possess awareness of AMR effects and AMS principles, systemic barriers hinder translation into consistent practice. These findings highlight the need for integrated AMS interventions that combine guideline dissemination, laboratory capacity strengthening, routine ward-level antibiogram feedback, enforcement of SAP duration standards, and institutionalized audit-and-feedback systems. Future research should evaluate the impact of targeted quality improvement interventions on reducing prolonged SAP and increasing culture-guided therapy, as well as assess behavioural and structural determinants of change over time. Programmatically, embedding AMS within surgical workflow, aligning pharmacy and laboratory systems, and addressing IPC gaps may be critical to interrupting the cycle between empirical antibiotic escalation and rising AMR. Ultimately, sustained improvements in antibiotic use will require coordinated investment in both human and infrastructural capacity to ensure that AMS awareness translates into measurable prescribing change.

## Conclusions and recommendations

This study demonstrates that inpatient antibiotic use in adult surgical wards of MNRH is high and characterized by near universal SAP, which is prolonged, with greater reliance on watch category rather than Access category antibiotics under the WHO AWaRe framework. These prescribing patterns are occurring within a context of documented AMR, limited culture guided therapy, suboptimal UCG adherence and fragmented AMS structures. While HCWs demonstrate conceptual awareness of AMS principles, systemic barriers such as laboratory constraints, drug availability challenges and limited audit-feedback systems impede translation of knowledge into consistent rational prescribing. Addressing these challenges requires coordinated structural, behavioural and institutional interventions rather than knowledge-based training alone.

Limiting duration of SAP to single dose in 24 hours for eligible procedures should be prioritized through ward level documented SOPs and routine prescription monitoring. Antibiotic prescribing should be re-balanced towards Access agents in alignment with WHO AWaRe targets, supported by dissemination of ward specific antibiograms to guide empirical choices. Institutionalized audit and feedback mechanisms including PPS, stewardship rounds, and prescription audits should be embedded within routine surgical workflow to enhance accountability. AMS responsibilities should be formalized with protected time and multidisciplinary HCW engagement involving surgeons, microbiologists, IPC teams and nurses. Finally, future research should evaluate effectiveness of targeted interventions on reducing prolonged SAP, increasing culture utilization and shifting AWaRe distribution overtime, while also examining cost effectiveness and sustainability of integrated AMS programs in similar settings. Collectively, these actions could translate stewardship awareness into measurable, sustained improvements in antibiotic use and resistance containment.

## Supporting information

S1 Fig Data collection process for point prevalence survey. (TIF)

S1 Table Sociodemographic characteristics of AMR survey participants. (DOCX)

S2 Fig Sample types processed for culture and sensitivity testing. (TIF)

S2 Table Sociodemographic characteristics of qualitative participants. (DOCX)

S3 Table Joint display showing integration of quantitative and qualitative results. (DOCX)

S1 File Point prevalence survey form used for data abstraction during this study. (PDF)
