# Supplementary material for: Patterns of inpatient antibiotic use and antimicrobial resistance in the surgical wards of a Ugandan tertiary hospital: A mixed methods study
Source: PLoS One. 2026 Jul 24;21(7):e0352983. doi: 10.1371/journal.pone.0352983 (PMC13399451; doi:10.1371/journal.pone.0352983)
Supplement: S1 Table — (DOCX) [file pone.0352983.s002.docx]

**S1 Table**

| **Characteristic (N=480)** | **Frequency n(%)** |
| --- | --- |
| **Median Age in years (IQR)** | 38 (28-53.5) |
| **Sex** |  |
| Female | 151 (31.5) |
| Male | 329 (68.5) |
| **Ward** |  |
| General and Gastrointestinal surgery | 137 (28.5) |
| Orthopaedic and Trauma surgery | 216 (45.0) |
| Neurosurgical and Spine surgery | 86 (17.9) |
| Burns, Plastics and Reconstructive surgery | 1(0.2) |
| Cardiothoracic and other specialized surgery | 40 (8.3) |
