# Supplementary material for: Patterns of inpatient antibiotic use and antimicrobial resistance in the surgical wards of a Ugandan tertiary hospital: A mixed methods study
Source: PLoS One. 2026 Jul 24;21(7):e0352983. doi: 10.1371/journal.pone.0352983 (PMC13399451; doi:10.1371/journal.pone.0352983)
Supplement: S2 Table — (DOCX) [file pone.0352983.s004.docx]

**S2 Table**

| **Characteristic** | **Frequency (N=29)** |
| --- | --- |
| **Median Duration in service in years (IQR)** | 3.5 (3-29) |
| **Cadres** |  |
| Resident doctors | 12 |
| Nurses | 12 |
| Pharmacists and Specialist Surgeons | 5 |
| **Gender** |  |
| Male | 18 |
| Female | 11 |
