# Supplementary material for: Patterns of inpatient antibiotic use and antimicrobial resistance in the surgical wards of a Ugandan tertiary hospital: A mixed methods study
Source: PLoS One. 2026 Jul 24;21(7):e0352983. doi: 10.1371/journal.pone.0352983 (PMC13399451; doi:10.1371/journal.pone.0352983)
Supplement: S3 Table — (DOCX) [file pone.0352983.s005.docx]

**S3 Table**

| **Quantitative Finding** | **Corresponding Qualitative Sub-Theme** |
| --- | --- |
| 54.1% utilization of Watch versus 45.9% utilization of Access antibiotics. | Preference for broad spectrum ‘big-guns’, early escalation in severe or uncertain cases and the belief that broader agents were more effective and more reliable. |
| 48.2% UCG compliance, predominantly empirical antibiotic use (96.4%) | Absence of documented hospital or ward specific SOPs for antibiotic prescription, reactive rather than routine culture testing, laboratory delays and reagent stock outs. |
| Mostly prescribed antibiotics were Ceftriaxone (31.7%), for empirical therapy. | Antibiotics were selected based on availability, affordability, familiarity from past experiences, perceived effectiveness and reliance on inherited prescribing norms. |
| Prescription of antibiotics for SPP (91.7%), with 93.4% of these receiving multi-day prophylaxis | Prolonged SPP was due to overcrowding, hygiene concerns, lack of trust in IPC practices and fear of contamination. |
| AMR survey showed high prevalence of *E.coli* and *S.aureus,* with high levels of resistance to Ceftriaxone (≥80%) | Lack of hospital-based AMR surveillance and use of past Microbiology data as well as Awareness of AMR and AMS but lack of implementation. |
