# Supplementary material for: Patterns of inpatient antibiotic use and antimicrobial resistance in the surgical wards of a Ugandan tertiary hospital: A mixed methods study
Source: PLoS One. 2026 Jul 24;21(7):e0352983. doi: 10.1371/journal.pone.0352983 (PMC13399451; doi:10.1371/journal.pone.0352983)
Supplement: S1 File — (PDF) [file pone.0352983.s006.pdf]

Point Prevalence Survey form- Patient form.

Hospital Code ________________________ Ward code ___________________________ Patient code __________________________

Patient demographics.

Admission Date	yyyy-mm-dd	Surgery Since Admission	Yes / No / UNK
Central Vascular Catheter	Yes / No / UNK	Peripheral Vascular Catheter	Yes / No / UNK
Urinary Catheter	Yes / No / UNK	Intubation	Yes / No / UNK
Patient on antibiotic	Yes / No	Number of antibiotics.	

Underlying infectious diseases (Optional)

Malaria status	Yes / No / UNK	Tuberculosis status	Yes / No / UNK
HIV status	Yes / No / UNK	HIV on ART	Yes / No / UNK

Comorbidities (optional)

Malnutrition status	Yes / No / UNK	COPD status	Yes / No / UNK

Hospitalization (optional)

Transfer From Hospital	Yes / No / UNK	Transfer From Non Hospital	Yes / No / UNK
Hospitalisation 90 Days	Yes / No / UNK		

Point Prevalence Survey form- Patient form.

Hospital Code ________________________ Ward code ___________________________ Patient code __________________________

Surgery variable (Optional, Only if Surgery Since Admission = Yes)

Type Surgery Since Admission	M / NHSN / UNK

Additional Comments:

Indications.- *Core variables.*

Indication Counter	1	2	3	4
Indication Type	HAI / CAI / SP / MP /	HAI / CAI / SP / MP /	HAI / CAI / SP / MP /	HAI / CAI / SP / MP /

If IndicationType is SP:

Surg.Proph.Duration		SP1 / SP2 / SP3	SP1 / SP2 / SP3	SP1 / SP2 / SP3
Surg.Proph.Site Diagnosis	Annex X	Annex X	Annex X	Annex X
StartDateTreatment	Annex IV	Annex IV	Annex IV	Annex IV
ReasonInNotes	yyyy-mm-dd	yyyy-mm-dd	yyyy-mm-dd	yyyy-mm-dd
CultureSampleTaken	YES / NO	YES / NO	YES / NO	YES / NO
If Culture Sample taken is	Yes / No / UNK	Yes / No / UNK	Yes / No / UNK	Yes / No / UNK

If culture sample take is YES, then proceed to Microbiology Form

Point Prevalence Survey form- Patient form.

Hospital Code ______________________ Ward code _________________________ Patient code ________________________

Additional Comments:

Antibiotics- *Core variables*

Antibiotic Counter	Indication Counters	Antibiotic Notes Name	Antibiotic INN Name	Antibiotic WrittenInINN	StartDate Antibiotic	UnitDose	UnitDoses Combination	UnitDose MeasureUnit	UnitDose Frequency	Administration Route
1				YES NO	yyyy-mm-dd			MG G IU MU		O P I R
2				YES NO	yyyy-mm-dd			MG G IU MU		O P I R
3				YES NO	yyyy-mm-dd			MG G IU MU		O P I R
4				YES NO	yyyy-mm-dd			MG G IU MU		O P I R
5				YES NO	yyyy-mm-dd			MG G IU MU		O P I R
6				YES NO	yyyy-mm-dd			MG G IU MU		O P I R

Point Prevalence Survey form- Patient form.

Hospital Code ________________________ Ward code ___________________________ Patient code __________________________

Antibiotics: *Optional variables*

Antibiotic Counter	PrescriberType	Parenteral Type	OralSwitch	Number MissedDoses	MissedDoses Reason	Guidelines Compliance	TreatmentType
1	SS<br>SHO<br>MO<br>ID<br>CO	IM<br>IV-B<br>IV-C<br>IV-E<br>O	YES<br>NO<br>UNK		S<br>P<br>O<br>M<br>UNK	Y<br>N<br>NA<br>NI	D<br>E
2	SS<br>SHO<br>MO<br>ID<br>CO	IM<br>IV-B<br>IV-C<br>IV-E<br>O	YES<br>NO<br>UNK		S<br>P<br>O<br>M<br>UNK	Y<br>N<br>NA<br>NI	D<br>E
3	SS<br>SHO<br>MO<br>ID<br>CO	IM<br>IV-B<br>IV-C<br>IV-E<br>O	YES<br>NO<br>UNK		S<br>P<br>O<br>M<br>UNK	Y<br>N<br>NA<br>NI	D<br>E
4	SS<br>SHO<br>MO<br>ID<br>CO	IM<br>IV-B<br>IV-C<br>IV-E<br>O	YES<br>NO<br>UNK		S<br>P<br>O<br>M<br>UNK	Y<br>N<br>NA<br>NI	D<br>E
5	SS<br>SHO<br>MO<br>ID<br>CO	IM<br>IV-B<br>IV-C<br>IV-E<br>O	YES<br>NO<br>UNK		S<br>P<br>O<br>M<br>UNK	Y<br>N<br>NA<br>NI	D<br>E
6	SS<br>SHO<br>MO<br>ID<br>CO	IM<br>IV-B<br>IV-C<br>IV-E<br>O	YES<br>NO<br>UNK		S<br>P<br>O<br>M<br>UNK	Y<br>N<br>NA<br>NI	D<br>E

***Microbiology results** (Microbiology data refers to any culture & susceptibility result from a relevant clinical sample. Screening samples should not be reported).*

Specimen 1: Specimen Type [Annex VII]

Culture Result [NA / Pos / Neg]

If Culture Result is Pos:

Microorganism: 1 [annex VI] 2 [annex VI] 3 [annex VI]

Antibiotic Susceptibility Test Results

1 [Yes / No / UNK] 2 [Yes / No / UNK] 3 [Yes / No / UNK]

Resistant Phenotype (Only if Antibiotic Susceptibility Test Results is Yes)

1 [annex VIII] 2 [annex VIII] 3 [annex VIII]

Specimen 2: SpecimenType [Annex VII]

Culture Result [NA / Pos / Neg]

If Culture Result is Pos:

Microorganism: 1 [annex VI] 2 [annex VI] 3 [annex VI]

Antibiotic Susceptibility Test Results

1 [Yes / No / UNK] 2 [Yes / No / UNK] 3 [Yes / No / UNK]

Resistant Phenotype (Only if Antibiotic Susceptibility Test Results is Yes)

1 [annex VIII] 2 [annex VIII] 3 [annex VIII]

Specimen 3: SpecimenType [Annex VII]

Culture Result [NA / Pos / Neg]

If Culture Result is Pos:

Microorganism: 1 [annex VI] 2 [annex VI] 3 [annex VI]

Antibiotic Susceptibility Test Results

1	Yes / No / UNK	2	Yes / No / UNK	3	Yes / No / UNK

Resistant Phenotype (Only if Antibiotic Susceptibility Test Results is Yes)

1	annex VIII	2	annex VIII	3	annex VIII

Specimen 4: SpecimenType: Annex VII

Culture Result: NA / Pos / Neg

If Culture Result is Pos:

Microorganism:

1	annex VI	2	annex VI	3	annex VI

Antibiotic Susceptibility Test Results

1	Yes / No / UNK	2	Yes / No / UNK	3	Yes / No / UNK

Resistant Phenotype (Only if Antibiotic Susceptibility Test Results is Yes)

1	annex VIII	2	annex VIII	3	annex VIII
